# ‘I take my tablets with the whiskey’: A qualitative study of alcohol and medication use in mid to later life

**DOI:** 10.1371/journal.pone.0205956

**Published:** 2018-10-18

**Authors:** Catherine Haighton, Jess Kidd, Amy O’Donnell, Graeme Wilson, Karen McCabe, Jonathan Ling

**Affiliations:** 1 Department of Social Work, Education and Community Wellbeing, Northumbria University, Newcastle upon Tyne, United Kingdom; 2 Institute of Health and Society, Newcastle University, Newcastle upon Tyne, United Kingdom; 3 Reid School of Music, University of Edinburgh, Edinburgh, United Kingdom; 4 School of Nursing and Health Sciences, University of Sunderland, Sunderland, United Kingdom; King's College London, UNITED KINGDOM

## Abstract

**Background:**

Concurrent alcohol and medication use can result in significant problems especially in mid to later life. Alcohol is often used instead of medication for a number of health-related conditions. This novel qualitative study explored concurrent alcohol and medication use, as well as the use of alcohol for medicinal purposes, in a sample of individuals in mid to later life.

**Methods:**

Twenty-four interviews (12 men/12 women, ages 51–90 years) and three focus groups (n = 27, 6 men/21 women, ages 50–95 years) from three branches of Age UK and two services for alcohol problems in North East England.

**Results:**

Older people in this study often combined alcohol and medication, frequently without discussing this with their family doctor. However, being prescribed medication could act as a motivating factor to stop or reduce alcohol consumption. Participants also used alcohol to self-medicate, to numb pain, aid sleep or cope with stress and anxiety. Some participants used alcohol to deal with depression although alcohol was also reported as a cause of depression. Women in this study reported using alcohol to cope with mental health problems while men were more likely to describe reducing their alcohol consumption as a consequence of being prescribed medication.

**Conclusions:**

As older people often combine alcohol and medication, health professionals such as family doctors, community nurses, and pharmacists should consider older patients’ alcohol consumption prior to prescribing or dispensing medication and should monitor subsequent drinking. In particular, older people should be informed of the dangers of concurrent alcohol and medication use.

## Introduction

Concurrent alcohol and medication use can result in significant problems especially in mid to later life (50 years and over)[1]. When alcohol is combined with nonsteroidal anti-inflammatory drugs it can cause stomach bleeding, gastric inflammation and liver damage[2]. Alcohol also enhances the sedative effects of antidepressants, antihistamines, barbiturates, muscle relaxants, benzodiazepines and opioids. This can have serious consequences such as falls, accidents and even death[2].

Older people are more likely to be at risk of alcohol-related adverse drug reactions because they are more likely to use multiple alcohol-interactive drugs[2]. The average person aged 50 years and over takes at least four medications a day[3]. In addition, alcohol consumption has recently increased among older age groups in many developed countries including the United States, Australia, and many parts of the European Union[4–7]. In the UK, older people tend to drink more frequently than younger people[8] with 17% of people aged over 50 drinking alcohol four or more times each week[9]. In addition, while older people might consume less alcohol overall than younger people[8], 17% of people aged over 50 are at increasing risk and 3% are at high risk of developing alcohol use disorders or dependence[9].

Research has shown that adults in mid to later life consume alcohol for a number of different reasons which can be categorised as either positive or negative reinforcement[10]. While stressful life events, such as bereavement or retirement, may trigger late-onset drinking in some, this is not the case for all[11, 12]. Alcohol use has been associated with self-medication for both physical and mental health problems as well as insomnia and has also been linked to boredom, loneliness, isolation and homelessness[11]. However, the direction of causality in the relationship between alcohol use and many of these factors is often in doubt[10]. Older people also report consuming alcohol for positive reasons such as enjoyment and socialisation[11, 13–14].

A number of systematic reviews and quantitative studies have shown that combined alcohol and medication use is common in older people[15–23]. A survey of Irish adults aged 60 years and over has shown that 60% of people using alcohol-interactive drugs also reported alcohol use[24]. A similar study of older adults aged 65 years and over in Finland reported 63% of those taking medication also used alcohol[22]. In the United States research has reported rates of combined alcohol and alcohol-interactive drug use ranging from 19% to 78% in older people aged 65 years and over[2, 23].

Alongside concurrent alcohol and medication use, alcohol itself is often used instead of medication for a number of health-related conditions[25, 26]. The extent of drinking for medicinal purposes has been quantified in a small number of studies in the United States, Canada and Finland[25–28]. Research in Finland, of people aged over 75 years, revealed that 40% reported using alcohol for medicinal purposes. The main reasons cited were heart and vascular disorders (38%), sleep disorders (26%) and mental health problems (23%)[26]. Chronic pain has also been associated with the use of alcohol in older people[27, 28]. Research, mainly from the United States, has revealed associations between problem drinkers and severity of pain[27], and suggests that those older individuals who have more painful medical conditions also have more frequent drinking problems[28].

However, whilst there is reasonable quantitative evidence on the prevalence of concurrent alcohol and medication use, and drinking for medicinal purposes in mid to later life, there is a lack of understanding of the views and experiences of older people themselves on this issue. Moreover, previous studies have been based outside the UK, despite the fact that evidence suggests the UK has both an aging population and high rates of heavy drinking amongst this population group. This lack of both qualitative and quantitative research in the UK and the limits of generalising research from other cultural contexts, means there is a real need for UK-based research in this area. This paper reports the first UK-based, qualitative study examining experiences of and attitudes to concurrent alcohol and medication use, as well as the use of alcohol for medicinal purposes, in a sample of individuals in mid to later life.

## Methods

The study involved qualitative interviews and focus groups with a purposive sample of people aged 50 years and over. Ethical approval was issued by Newcastle University Research Ethics Committee (application no. 000224/2009). Participants provided written informed consent to participate in this study and for data to be published. In line with the terms of consent to which participants agreed, the data are not publicly available.

From an epistemological stance (the set of assumptions that help explain the nature of the world)[29] this research is situated within the interpretivist paradigm. Interpretivism is associated with the understanding of complex and often changing entities. Interpretivists assert that there is no such thing as a single objective reality. Rather, there are multiple constructed realities, because different people are likely to experience the world in differing ways. The interpretivist approach delivers a contextualised, rich, detailed account of human experiences[30]. In order to gain this depth of understanding, a range of qualitative methods were employed. We combined one-to-one interviews with focus groups in order to compare individual accounts with perspective that have been socially negotiated. Triangulation of data from these two sources was intended to situate the individual beliefs within the social discourse likely to have shaped their production.

Twenty-four qualitative interviews (12 men, 12 women) were conducted between 19/11/09 and 15/03/10. Sample size was determined by reaching data saturation where the research team deemed no new themes to have emerged in three consecutive interviews[31]. Purposive sampling (a non-random method of ensuring that particular categories of cases within a sampling universe are represented in the final sample)[32] aimed to recruit both sexes and represent a broad range of ages and self-reported drinking practices. This sampling approach was intended to reflect those who might request help or support from Age UK, the UK’s leading charity for older people (http://www.ageuk.org.uk/). Age UK provides services and support at a national and local level to “inspire, enable and support anyone over the age of 50 years”[33].

Three branches of Age UK and two services for alcohol problems, covering a wide geographical area, distributed research information leaflets to clients aged 50 and over with experience of drinking alcohol. Staff from the recruiting organisations invited clients to consider participating in an interview, answered any questions they had about the research and asked those who were interested to complete a consent form.

All potential participants were contacted by telephone by one of the authors (GW) to arrange an interview. As the initial sample appeared to consist of a large proportion of participants who described themselves as recovering dependent drinkers, strategic ‘snowballing’ was used to add further interviewees. This involved existing study participants recruiting future participants, who were not using services and therefore not recovering dependent drinkers, from among their acquaintances. In total, nine participants were recruited via Age UK, 13 via services for alcohol problems and two via strategic snowballing. Considerable diversity was ultimately achieved in terms of gender, age, social situation and level of alcohol consumption (see Table 1).

**Table 1:** Interviewee characteristics.

Interviewee number	Age	Sex	From interview: self-reported drinking status /behavior	From interview: lives with
1	61	m	Recovering dependent drinker Abstinent for 2.5 years	Other residents
2*	59	f	Recovering dependent drinker Sensible drinker for 12 years	Adult child Adult child‘s partner Grandchild
3*	56	f	Dependent drinker	Husband Adult child
4*	61	m	Dependent drinker	Alone
5	52	m	Recovering dependent drinker Abstinent for 2 months	Alone
6	59	m	Recovering dependent drinker Abstinent for 4 weeks	Wife
7	57	m	Recovering dependent drinker Abstinent for 2 years	Wife
8*	74	m	3 litres whiskey per week	Alone
9	62	m	Previously 3–4 pints on 3–4 nights per week Abstinent for 6 months	Alone
10	60	m	Recovering dependent drinker Abstinent for 1 year	Alone
11	55	f	Recovering dependent drinker Abstinent for 9 weeks	Alone
12	51	f	Previously 3 litres cider + 2 cans per day Abstinent for 1 year	Husband Teenage children
13	68	m	Recovering dependent drinker Abstinent for 5 years	Unknown
14*	58	f	Previously 2 bottles spirits per weekend Reduced to occasional glass of wine for past 2 years	Alone
15*	65	m	Previously 13 pints beer per night Reduced to 2–3 pints per night for 1.5 years	Alone
16*	52	f	Reducing dependent drinker From bottles of spirits to 4 pints, 5 days a week	Husband Adult children
17*	70	f	Bottle of wine a day Abstinent while hospitalised only	Other residents
18*	78	f	Occasional minimal drinker	Other residents
19*	83	f	Occasional minimal drinker	Other residents
20*	90	f	Occasional minimal drinker	Other residents
21*	56	m	4–5 pints/night, 2 nights/week Reduced from previous levels	Partner & sons
22*	59	f	Previously a bottle a night for a period Reduced to glass or two of wine a night, not every night.	Partner
23*	58	F	4 vodka & tonics a night, twice a week	Partner
24*	72	M	4 pints beer every night, sometimes two gin and tonics	Wife

*Currently consuming alcohol

Three focus groups were facilitated between 15/03/10 and 19/10/10. Staff at the three branches of Age UK distributed research information leaflets to members with experience of drinking alcohol, inviting them to consider participating in a focus group. Staff answered any questions they had about the research and asked those who were interested to complete a consent form. The first group comprised 9 participants (1 man, 8 women, ages 79–95); the second group comprised 12 participants (5 men, 7 women, ages 50–85) and the third comprised 6 participants (all women, ages 51–76). Participants in each focus group were known to each other through their membership of Age UK. To encourage participation, focus group participants were not required to disclose personal details other than age and date of birth; these data were gathered on consent forms. At the groups, participants were invited by the facilitator (GW) to offer views in general rather than recounting personal experiences in front of others.

Interviews and focus groups were conducted by GW, a male, experienced post-doctoral researcher, lasted between 40 and 150 minutes, and took place either at individual respondent’s homes or the offices of participating organisations. The research team prepared topic guides (see supplementary files) to initiate or return discussion to the research topics; drinking in later life, experiences of service use and support, and how support could be improved. In addition, participants were specifically asked whether they were taking any prescription medications, whether this was affected by alcohol and if so how, and whether their doctor had discussed this with them. The interviewer was not previously known to participants and introduced himself as a researcher with an interest in alcohol use in later life. Interview and focus group data were audio recorded, transcribed verbatim, anonymised and loaded into NVivo qualitative software, version 10[34]. Data were analysed using a grounded approach[35–36] which operates well within the interpretivist paradigm and starts with the qualitative data collected. As the data are reviewed, repeated concepts become apparent. As more data are collected and re-reviewed, these concepts can be grouped into themes. The original position of Strauss and Corbin[35] was one of an objective researcher who tries to represent an external reality as accurately as possible. Analysis involved repeatedly reading transcripts and identifying emerging themes; early analysis informed later interviews and focus groups. Authors were aware that the interviewer was part of the research process and, along with those being researched, brought with him concepts, ideas, theories, values and experiences. Therefore themes were refined through discussion amongst all the authors with consideration of divergent cases in order to provide a full account of the data that recognises and explores how demographic factors will have shaped participants’ views. Focus group data were used to triangulate findings from individual interviews. In the results section below we report findings from individual interviews, then consider how the focus group data inform or challenge these themes. The original study aimed to elucidate the views of older individuals about alcohol consumption, health, and well-being in order to inform future targeted prevention in this group [11, 37]. This paper is not exhaustive in its presentation of the analysis; rather it focuses on specific themes (with each subheading representing a distinct theme) which emerged from the data relating to alcohol and medication.

## Results

Four distinct themes emerged from the data: drinking and using medication regardless of potential consequences; health professionals being unaware of alcohol use; reducing or stopping alcohol consumption because of medication; and using alcohol to self-medicate. Each of these themes will be discussed below.

### Drinking and using medication regardless of potential consequences

#### Individual interviews

Several of the participants continued to drink alcohol even though they were using medication. One woman explained that doctors ‘don’t know what I’m going through’ and said she continued to drink even though she was on medication for bi-polar disorder (Interviewee 2, f, 59, Recovering dependent drinker—sensible drinker for 12 years). A number of participants reported that they continued drinking and using medication despite being aware of the consequences because their desire for alcohol was so strong that they would select alcohol over medication.

No, no. I think if an alcoholic had to choose between medication and his alcohol they would probably do without medication. Alcohol is a very, very, powerful addiction. It is a very, very, very, difficult to cure something with alcohol addiction (Interviewee 13, m, 68, Recovering dependent drinker—abstinent for 5 years)

Some of the participants who reported continuing to drink while taking medication said they did so because they were not troubled by the side effects. One woman described how her ‘body is used to alcohol’ and that she did not feel the side effects of combining it with medication because she had a ‘high tolerance to alcohol’ (Interviewee 16, f, 52, Reducing dependent drinker—from bottles of spirits to 4 pints, 5 days a week). Some participants went on to explain that they even used alcohol to take their medication.

I don’t think it makes any difference. I’ll come down on a morning after I’ve taken a bath upstairs. I hardly touch the drink upstairs. I’ll bring it down. I take my tablets with the whiskey. (Interviewee 8, m, 74, 3 litres whiskey per week)

However, some participants continued to drink and take their medication despite experiencing the side effects. The strength of some participants’ desire to continue drinking, despite the risks of combining alcohol with their medication, underlines the complex and dynamic relationship between the pleasure and harm of alcohol use.

I must admit a couple of Ativan and a pint of Strongbow and that used to affect me. So really I’ve just made a right arse of myself. When you’re mixing them you get high on the Ativan and the booze, but when you come down you know maybe the next day or into the next day, you get a bigger downer. (Interviewee 15, m, 65, Previously 13 pints beer per night—reduced to 2–3 pints per night for 1.5 years)

#### Focus groups

Focus group participants also talked about continuing to drink despite using medication, but referred to friends and family rather than to themselves.

There is some people who will see maybe that on a medication bottle–“Avoid alcohol”, and just think it’s there for someone else but not for me and drink anyhow. (Focus Group 2, m)

I think with the older people there’s a lot of them on medication as well and they’re still drinking. (Focus Group 3, f)

Focus group participants also reported some people’s desire for alcohol being so strong that they would rather drink than take their medication, but again this was not in relation to their own drinking behaviour.

If somebody drinks, they won’t take their medication, they would rather drink. I know people, they get their tablets off the Doctor and they won’t take them. They’d rather drink. (Focus Group 2, f)

Focus group participants also talked of relatives experiencing side effects from combining alcohol with medication.

I mean my father-in-law is a good example of that: he’s on medication for various things and he drinks. And it makes him worse and it makes his mood worse. (Focus Group 3, f)

There was no mention at focus groups of individuals using alcohol to take their medication. At focus groups people tended to describe the experiences of others rather than themselves suggesting that while individuals may justify their own co-use to themselves, the social norm is for this to be seen as a negative behaviour. There was no apparent difference between men and women in drinking and using medication, regardless of potential consequences.

The potential consequences of continuing to drink alcohol while using medication were generally framed negatively within this theme. However, given the social connection, relaxation and other benefits of alcohol use that have been reported amongst older adults in other studies, the reasons for continuing to drink while using medication may have been positive ones such as preventing social isolation, for pleasure, to aid sleep or gain temporary relief from pain. This will be discussed further under the theme using alcohol to self-medicate.

### Health professionals being unaware of alcohol use

#### Individual interviews

In a number of instances participants suggested that their family doctors were unaware that they were consuming alcohol. When asked whether their family doctor enquired about alcohol, one woman stated that her family doctor had never asked her (interviewee 11, f, 55, Recovering dependent drinker—abstinent for 9 weeks). Another man explained that there had never been any mention of drinking between him and his family doctor (interviewee 15, m, 65, Previously 13 pints beer per night—reduced to 2–3 pints per night for 1.5 years). One man revealed that if his family doctor had told him of the dangers of combining alcohol and medication his drinking habits might be different.

No, because if I was being warned; if somebody warned me, if somebody in the medical profession warned me that I was doing something medically dangerous and that you increase; and that’s why I stopped smoking, because it increases your chance of having a heart attack. (Interviewee 21, m, 56, 4–5 pints/night, 2 nights/week—reduced from previous levels)

#### Focus groups

Once again focus group participants all talked about friends and family, rather than themselves. Focus group participants all reported these people having been advised by their family doctor not to consume alcohol alongside their medication. This contrasting view could, once again, be as a consequence of focus group participants making assumptions for others rather than talking from personal experience.

I’ve got a friend who had lung cancer and she takes certain drugs and they tell her to avoid alcohol, and she drinks. Now after she’s had a drink, she always two or three days when she’s not well. Now we’ll say to her, why do you do it, you know, if that’s telling you; she’s had, to me, the biggest scare in her life, and getting over it by having a lung taken away, with lung cancer, and she’s now sort of 15, 16 years on, why would you want to make yourself ill for two or three days, by going out and having a drink, just because that’s not there for you, for that night when you go out and have the alcohol. (Focus Group 2, m)

Again, focus group participants reported ‘worst-case’ scenarios to emphasise that they recognised the correct attitude to be that co-use of alcohol and medication is negligent of health. There was no apparent difference between men and women regarding whether a health professional was aware of their combined alcohol and medication use.

### Reducing or stopping alcohol consumption because of medication

#### Individual interviews

Some of the participants reported reducing or stopping alcohol consumption as a consequence of being prescribed medication. Many participants understood the dangers of concurrent alcohol and medication use and were aware of the adverse reactions that could occur when the two are combined. This awareness came, for some, from reading the labels on the medication bottles and for others from their family doctor. One woman described herself as ‘an intelligent alcoholic’. She acknowledged the risks of combining alcohol and medication and explained that while she was still drinking it was at a fraction of her previous rates.

Because half of us that are on tablets are not supposed to drink. And we’re aware of that, we know that. I think that’s why I’m not an alcoholic anymore, because I know my medication takes me to a certain level. Alright this weekend I’ve been down, but that’s how my bipolar works–I’m either up, or I’m down, or I’m in the middle. (Interviewee 2, f, 59, Recovering dependent drinker—sensible drinker for 12 years)

Another participant described how he had to stop drinking, partly through necessity rather than choice, as his family doctor would not prescribe medication while he was drinking heavily because of the adverse side effects that could occur even with mild medications.

They have to say ‘here–here’s medication for your anxiety–anti-depressants’ and all that. It’s impossible for you to get them at the time you’re drinking alcohol because it could kill you. Because personally I felt I was getting let down by the GPs–they wouldn’t give me nothing for my anxiety and stress. Because of alcohol you can’t get treatment with nothing really. Even if you have toothache and you’re an alcoholic and drinking they can’t give you nothing. (Interviewee 5, m, 52, Recovering dependent drinker—abstinent for 2 months)

Some participants changed their drinking behaviour because of negative previous experiences of combining alcohol and medication. One woman talked of suffering from a ‘horrible hangover’ after drinking alcohol with her medication (Interviewee 16, f, 52, Reducing dependent drinker—from bottles of spirits to 4 pints, 5 days a week) and a man actually had his medication reduced because of the effects of combining it with alcohol.

Yes–it does, but they reduced it because I was walking funny. It’s supposed to give you alarm bells you know. But as I said, you get that much pressure you’ve got to do something. Whether I made the right decision or not, I don’t know. I find it’s not hard to pack in drinking, but packing in smoking would be harder for me. (Interviewee 9, m, 62, Previously 3–4 pints on 3–4 nights per week—abstinent for 6 months)

On the other hand, some interviewees simply took the advice of their family doctor and were not willing to wait and experience the consequences of combining the two. These participants trusted their family doctors’ expertise so much so that they would simply do what was advised.

Yeah I do have medication, I take medication for a hiatus hernia and no, it has no effect on my thoughts of drinking at all, Because I don’t; maybe I’m kidding myself here, is half a glass too much, or a glass too much of a night, I don’t consider myself to be a heavy drinker. I suppose if I had a new medication, that I didn’t know [how] it would affect [me], and the doctor had specifically said to me, ‘You mustn’t drink with this’, then I just wouldn’t have a drink. (Interviewee 22, f, 59, Previously a bottle a night for a period—reduced to glass or two of wine a night, not every night)

With the majority of participants reporting one or more health problems, multiple medications were not uncommon. In some instances, alcohol was seen as counteracting the beneficial properties of the medication. One woman stated that continuing to drink while on her medication for chronic pancreatitis would increase her risk of developing pancreatic cancer ‘100-fold’ (interviewee 12, f, 51, Previously 3 litres cider + 2 cans per day—abstinent for 1 year). One man talked about the negative effects of alcohol on medication.

What you’ll find is, with a lot of people, right, when they’re drinking, they’re depressed. They’ll initially go to the doctors because they’re depressed, they get the Valium or whatever else you want to take, antidepressants but then you still keep drinking. Any drug or nine out of ten have no effect on you if you’re drinking. You take antibiotics; them antibiotics aren’t going to work. You take alcohol when you’re taking antidepressants, them antidepressants are…you might as well flush them down the toilet. So you do get that situation where if you’re drinking, medication’s not gonna work and I would image that works with whether you’re taking drugs, drugs for your arthritis or whatever, I don’t know, but usually they say if you’re taking alcohol, it tends to negate your…I mean I’m not a medical man, I’m only going by…I know antidepressants don’t work if you’re taking alcohol. (Interviewee 7, m, 57, Recovering dependent drinker—abstinent for 2 years)

#### Focus groups

During focus groups discussions, some participants also reported that they themselves had either reduced or stopped drinking alcohol as a direct consequence of being prescribed medication. In addition, however, those participants who had not been prescribed medication reported that they would not combine alcohol with prescribed medication if alcohol was contraindicated.

Well a lot of medication usually say ‘do not mix with alcohol’ so I wouldn’t anyway (Focus Group 1, f)

In contrast to the themes reported above, focus group participants talked about their own experiences, rather than the experiences of others, when discussing reducing or stopping drinking as a result of being prescribed medication. However, this may be due to the fact that this was seen as a positive and acceptable change in behaviour. Once again, at focus groups, participants presented what appears to be the most socially acceptable type of behaviour in response to being prescribed medication which could interact with alcohol.

While participants did not refer directly to any gender differences in reducing or stopping alcohol consumption because of medication, by examining the characteristics of the participants who reported that they had stopped drinking due to medication it was revealed that the majority were men.

### Using alcohol to self-medicate

#### Individual interviews

Some participants who reported continual heavy drinking while their health was deteriorating declared that they did so as they were using alcohol to self-medicate. One man who described having multiple pains in his joints due to arthritis reported that as soon as he started drinking the pain left. He used words such as ‘numbing’ as he was aware that when the effects of the alcohol wore off, the pain would return and it was therefore not a permanent solution (interviewee 14, f, 58, Previously 2 bottles spirits per weekend—reduced to occasional glass of wine for past 2 years). Another woman said that alcohol made her ‘happier’ with respect to her back pains as every time she felt in agony she would have a drink and the pain would stop (interviewee 17, f, 70, Bottle of wine a day—abstinent while hospitalised only). This use of alcohol as a pain medication was highlighted by others.

But it was Boxing Day. I was out and a friend grabbed me and gave me a bear hug, now he’s busted my ribs. You see, when I’m in pain I drink more–and that’s why I’m drinking more. (Interviewee 3, f, 56, Dependent drinker)

In other instances, individuals were directly substituting their medication for alcohol, most commonly for help with sleep. Instead of taking sleeping tablets, participants believed a glass of whiskey would have similar effects. One woman was skeptical of alcohol’s quality as an aid to sleep yet continued to use it perhaps because as it had become routine.

But you know, as I say, that’s the only time I think ‘oh, I’ll have a drop of warm hot whiskey before I go to bed’, hoping it’ll, you know, put me to sleep. But if I didn’t have it, it wouldn’t bother me. (Interviewee 20, f, 90, Occasional minimal drinker)

A number of participants also reported drinking to cope with mental health problems. Depression was the most commonly self-medicated disorder reported but alcohol was also used when dealing with panic attacks, bi-polar disorder, anxiety, stress or a combination of disorders.

I went through a bad stage of depression then and I was drinking a lot then…so I was off work obviously with anxiety and stress or whatever for 6 months. Then depression, the thing was I turned to drink to sort of shave the edges if you like–to put on not so much rose-coloured spectacles, but to shade reality and to get a bit out of reality to, I suppose, not facing things as they are. And depression, you know have a drink and then have another one or whatever. (Interviewee 12, f, 51, Previously 3 litres cider + 2 cans per day—abstinent for 1 year)

While some interviewees drank because of depression, other interviewees described alcohol as a cause of their depression. One man described how, after a session of drinking he ‘feels worthless’ and ‘has nobody to turn to’ which caused him to continue drinking (interviewee 7, m, 57, Recovering dependent drinker—abstinent for 2 years). For many this was an ongoing cycle without knowledge of what was cause and what was effect.

Because to me, depression and alcohol, they’re like which came first? The chicken or the egg? You know? (Interviewee 12, f, 51, Previously 3 litres cider + 2 cans per day—abstinent for 1 year)

Participants did not always ascribe to one or other view; depression could be seen as making one drink to gain short-term relief, even if they knew in the longer term it was likely to make them more depressed.

#### Focus groups

Focus group participants also described using alcohol to self-medicate. The majority described alcohol’s sedative properties as an aid to sleep, although a couple of participants mentioned using alcohol to cope with their depression. This like-minded behaviour is interesting given the earlier discourse at the focus group portraying the co-use of alcohol and medication as negligent to health.

But if for any reason I couldn’t get to sleep, even with the painkillers and chamomile tea I would get up and have a couple of sips of brandy. (Focus Group 1, f)

While the numbers of men and women reporting using alcohol to self-medicate did not vary substantially, a difference emerged in relation to mental health problems, with mainly women stating they used alcohol to cope with a range of issues from panic attacks to stress.

How many, how many older, older women have you seen in and they’ve said do you have a drink and they say erm well I’ll have a glass of whiskey sometimes for medicinal purposes. (Focus Group 3, f)

## Discussion

Older people in this study often combined alcohol and medication, frequently without discussing this with their family doctor. However, being prescribed medication could act as a motivating factor to stop or reduce alcohol consumption. Older people in this study also self-medicated, regularly using alcohol to numb pain, aid sleep or cope with stress and anxiety. Some participants used alcohol to deal with depression although alcohol was also reported as a cause of depression. Women in this study tended to report using alcohol to cope with mental health problems while men were more likely to describe reducing alcohol consumption as a consequence of being prescribed medication.

Other research has shown that depression, anxiety and other mental health problems are commonly seen in older individuals with alcohol dependence[38], with many older adults who develop depressive symptoms having a higher likelihood for day drinking[39]. One study revealed that while older people commonly drank alcohol to enhance positive daily experiences, they also drank to mask negative experiences. The study also reported that women had a tendency to drink more alcohol when stressed compared to men[40], which is consistent with findings from this study. Another interview-based study with community-dwelling older people reported that 23% cited using alcohol to deal with mental health problems[26].

However, the relationship between substance use disorders and mental health problems such as depression and anxiety is complex and the direction of causality is often in doubt. For example, does increased alcohol intake result from ‘self-medication’ in depression, or is the depression secondary to high levels of consumption? Davidson[41] reported that depression and alcohol dependence are frequently found to co-exist but the relationship between these disorders required further elucidation. His study tested several hypotheses related to the relevance of whether a diagnosis of depression was made before admission or after detoxification in an episode for those with alcohol dependence. For the episode of drinking which led to admission, a diagnosis of major depression was found in the majority of patients (67%). Once detoxification from alcohol took place, only a minority (13%) met criteria for major depression. He suggested therefore that depression is largely associated with the episode of drinking which led to admission in patients who are dependent on alcohol and may be due to the effect of chronic alcohol intoxication[41]. Alcohol consumption has also been linked to pain, with the majority of older adults who report drinking problems also reporting severe pain[34]. The “self-medication” hypothesis states that alcohol consumption is increased to combat rising levels of pain and has also been positively identified in younger adults[42]. Older problem drinkers in one study reported more severe pain, more disruption of daily activities due to pain, and more frequent use of alcohol to manage pain than older non-problem drinkers[27]. Other interview-based research has reported that 40% of older people used alcohol for medicinal purposes. This was equally common in women, and the most common reasons for use were reported to be heart and vascular disorders (38%) and sleep disorders (26%)[26]. The use of alcohol in aiding sleep was also reported by Sproule et al., revealing that 6% of older people chose alcohol as a non-prescriptive medication to help them sleep[25].

Findings from this study are consistent with the quantitative literature which suggests that the extent of combined alcohol and medication use among older people is widespread[2, 23]. However, this study further highlights that many people combine alcohol and medication despite knowing the potential consequences. Nevertheless, some participants in this study were unaware of the consequences of combining alcohol with their medication as they reported that their family doctors had never enquired about their use of alcohol. This is not surprising given that even when family doctors are encouraged to screen for alcohol problems they may both under-detect alcohol use disorders among older patients[43] and under-deliver health-promoting advice to them[44]. Furthermore, nurses report avoiding engagement with older people about alcohol use as they worry about depriving them of the social benefits of drinking[45–46].

Findings from this study are also consistent with the literature revealing that older people often use alcohol to self-medicate[25, 26] to manage pain[27, 28], aid sleep[25, 26] and to cope with mental health problems[26]. However, the qualitative nature of this study revealed that some older people continue to use alcohol to self-medicate even when they are skeptical of its medicinal properties. This study did indicate however that some older individuals will view being prescribed medication as a reason to reduce or stop their alcohol consumption, particularly when instructed by their family doctor to do so, through fear or experience of the adverse reactions. A large quantitative survey in the US found individuals who initiated antipsychotic and antineoplastic therapies were most likely to quit drinking[16]. However, some older individuals in this study rationalise their continued drinking and medication use on the grounds that they do not experience noticeable side effects.

This research contributes the first qualitative data regarding alcohol and medication use in mid to later life in the UK. The sample was restricted to a single region of the UK and with some characteristics of the sample over-represented (e.g. women and recovering dependent drinkers). Whilst attempts were made to recruit other types of drinkers we found higher-than-expected levels of enthusiasm among the treatment sample who appeared keen to share their experiences. In keeping with the assumptions of qualitative research, participant views expressed in interviews are recognised as having been intrinsically shaped by social influences. The specific use of focus groups to assess socially-negotiated versions of drinking in later life, and therefore what beliefs and attitudes might be seen as socially desirable in the social milieu of the individual interviewees, is a strength of this research. However, two of the focus groups contained participants from a broad age range, and given that focus groups are sensitive to power and social group dynamics, this might have affected participants’ willingness to express their views.

Triangulation of data appeared effective as similar themes arose from both interviews and focus groups although participants in the focus groups spoke of issues with alcohol and medication use as being experienced by family and friends rather than themselves. This lack of personal experience could however simply have been a consequence of recruiting focus group participants only via Age UK. As a result, focus group participants were more likely, than interview participants, to be moderate drinkers and this must be acknowledged as a limitation to triangulation. The negotiated social norm relating to combined alcohol and medication use was that it was not an acceptable form of behaviour, unlike using alcohol to self-medicate which was endorsed. This might be explained, at least in part, by the concept of ‘healthism’ or the imperative of health, which proposes an individual’s pursuit of health is a valuable activity that responsible individuals undertake [47]. When individuals engage in healthy practices they may feel good about themselves but when they fail to do so they may feel distressed or stigmatised (e.g. heavy alcohol consumption). Given that self-medication is a practice that is commonly engaged in and to some extent considered a normal way of attempting to address ailments it may be that using alcohol to self-medicate was an extension of these broader societal norms [48, 49]. Further qualitative research should explore these important findings in other geographical locations of the UK and internationally and with greater representation from male participants.

## Conclusion

There is a complex relationship between alcohol and medication use which is multi-faceted and related to other issues such as mental health. Our data revealed two main groups of participants. The first group was those participants who used alcohol with, or instead of, their medication. This group was knowledgeable about the contraindications but did not seem overly concerned about the potential risk. It is important therefore that health professionals including family doctors, community nurses, and pharmacists consider older patients’ alcohol consumption prior to prescribing or dispensing medication. Health professionals should also monitor older people who have been prescribed alcohol-interactive medication, for subsequent drinking[50]. Ultimately, health professionals need to address the underlying reasons for alcohol use such as mental health, pain, insomnia or dependence. The second group of participants was those who were unaware of alcohol-related adverse drug reactions and who would have benefitted from being informed of the dangers of concurrent alcohol and medication use. Overall evidence based guidelines regarding alcohol use in mid to later life are needed, especially in the UK, to support health professionals.
